# Viruses Seen by Our Cells: The Role of Viral RNA Sensors

**DOI:** 10.1155/2018/9480497

**Published:** 2018-04-30

**Authors:** Elias A. Said, Nicolas Tremblay, Mohammed S. Al-Balushi, Ali A. Al-Jabri, Daniel Lamarre

**Affiliations:** ^1^Department of Microbiology and Immunology, College of Medicine and Health Sciences, Sultan Qaboos University, P.O. Box 35, 123 Muscat, Oman; ^2^Centre de Recherche du CHUM (CRCHUM) et Faculté de Médecine, Université de Montréal, Montréal, QC, Canada

## Abstract

The role of the innate immune response in detecting RNA viruses is crucial for the establishment of proper inflammatory and antiviral responses. Different receptors, known as pattern recognition receptors (PRRs), are present in the cytoplasm, endosomes, and on the cellular surface. These receptors have the capacity to sense the presence of viral nucleic acids as pathogen-associated molecular patterns (PAMPs). This recognition leads to the induction of type 1 interferons (IFNs) as well as inflammatory cytokines and chemokines. In this review, we provide an overview of the significant involvement of cellular RNA helicases and Toll-like receptors (TLRs) 3, 7, and 8 in antiviral immune defenses.

## 1. Introduction

If a living organism wants to engage, control, and eliminate a pathogenic entity, it must first be able to detect it. At first, this simple yet elegant paradigm might seem easy enough to crack experimentally, but in retrospect, it has been a central research question for more than 50 years now.

From the pioneering studies to identify interferon- (IFN-) inducing compounds, to the discovery of Toll-like receptors (TLR), RIG-I-like receptors (RLR), and the cGAS-STING pathway, the quest to understand how pattern recognition receptors (PRR) recognized pathogen-associated molecular patterns (PAMP) has shed light on a complex network of signaling pathways that are spatially compartmentalized, mostly pathogen specific and highly/tightly regulated.

In this review, we will focus on the significant contribution of cellular RNA helicases and TLRs 3, 7, and 8 to antiviral immune defenses.

## 2. The Classical RNA Helicases of Antiviral Innate Immune Responses

In the wake of the discovery of TLRs, it was historically postulated that antiviral immunity was mediated via TLR3 because this membrane-anchored receptor was essential to trigger the production of type 1 IFNs and the activation of IFN stimulated genes (ISGs) when challenged with extracellular double-stranded RNA (dsRNA) poly(I : C), as a viral surrogate [1]. However, further investigation revealed that mouse TLR3−/− dendritic cells (BMDCs) can produce high levels of IFN*α* when stimulated with intracellular dsRNA suggesting the existence of another type of RNA sensor, beside the TLRs, that would survey the cytoplasmic space for pathogenic nucleic acids [2]. Further studies would identify RIG-I, MDA5, and LGP2; all RNA sensors of what is now known as the RLR signaling pathway.

**Figure 1:**
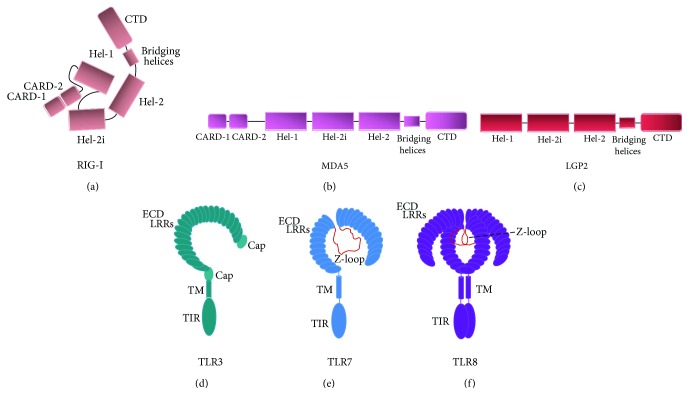
The structure of PRR implicated in detecting vRNA. RLRs are composed of a C-terminal domain (CTD), helicase domains (Hel), and two caspase-activation and recruitment domains (CARD-1, CARD-2) for (a) RIG-I and (b) MDA5, and only a CTD and helicase domain for (c) LGP2. TLRs 3, 7, and 8 are composed of an extracellular domain (ECD), a transmembrane (TM) domain, and a toll-interleukin 1 receptor (TIR) domain. The ECD contains 23 leucine-rich repeats (LRRs) for (d) TLR3 and 26 LRRs for (e) TLR7 and (f) TLR8. TLRs 7 and 8 have a Z-loop in the ECD. TLR8 exists as a dimer in the resting state.

**Figure 2:**
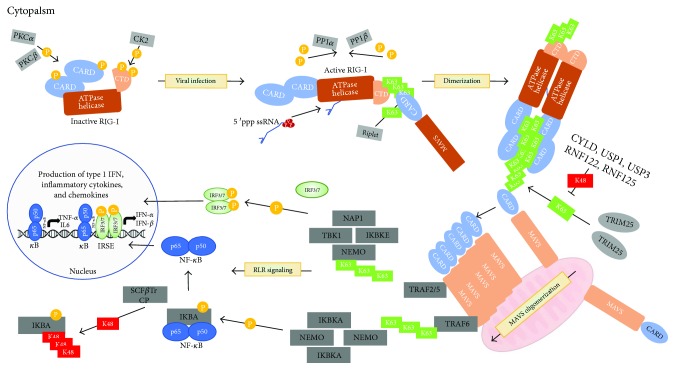
The pathways induced by RIG-I. Activation of RIG-I is regulated by many posttranslational modifications such as phosphorylation and ubiquitination. In resting cells, inactive RIG-I is kept in a close conformation by PKC*α*. PKC*β* and CK2 phosphorylate both CARDs and CTD. Upon viral infection, PP1*α* and PP1*β* dephosphorylate RIG-I to allow the binding of viral RNA within its ATPase-helicase domain which shifts RIG-I to an open conformation and allows the CTD to be ubiquitinated by Riplet. Once activated, TRIM25 allows for the recruitment of K63-polyubiquitin chains via TRIM25 which allow RIG-I dimerization and recruitment to the adaptor protein MAVS. To balance immune activation, CYLD, UPS1, UPS3, RNF122, and RNF125 actively antagonize RIG-I activation by the degradation of K63-polyubiquitin chains and a switch to K48-polyubiquitin chains that tag RIG-I for proteasome degradation. This interaction allows for the oligomerization of MAVS and the recruitment of regulatory subunits TRAF2, TRAF5, TRAF6, and NEMO. This signaling culminates with the phosphorylation of immune transcription factors via IKBKE, TBK1, and IKK protein kinases, leading to their nuclear translocation and production of type 1 IFN with subsequent expression of ISGs.

The retinoic acid-inducible gene I (RIG-I) was first identified as a cytoplasmic sensor that recognizes viral nucleic acids and triggers a signal to induce innate immune responses during viral infection [3]. The protein comprises two caspase-activation and recruitment domains (2CARDs) at the N-terminal region, an RNA helicase domain, and a C-terminal domain (CTD) (Figure 1(a)). In resting cells, the CTD suppresses the N-terminal 2CARDs that are responsible for the association with mitochondrial antiviral-signaling (MAVS) (also called IPS-1, CARDIFF, and VISA) and required for triggering downstream signaling (Figure 2) [4]. After recognition of intracellular virus-derived RNA (vRNA), the binding of the CTD to vRNA induces the conformational change of the RIG-I protein, resulting in the release of the 2CARDs and allowing the proteins to assemble along the vRNA and to form a nucleoprotein filament. The released 2CARDs form a tetramer structure [5] that functions as a core for CARD-containing MAVS aggregation on the outer membrane of the mitochondria. RIG-I activation is tightly regulated by posttranslational modifications (PTMs) such as phosphorylation and ubiquitination [6, 7]. In resting cells, CK2, PKC*α*, and PKC*β* protein kinases phosphorylate RIG-I, which keeps them in an inactive closed state to limit its activation (Figure 2) [8]. Upon viral infection, these PTMs are rapidly removed via two phosphatases (PP1*α* and PP1*β*) to shift RIG-I conformation into an active open state, which exposes its CARD domains and makes them available for subsequent ubiquitination [9]. Exposed CARDs are then targeted by TRIM25, Riplet, TRIM4, or MEX3C for K63 ubiquitin linkage, which is essential to allow its interaction with downstream adaptor protein MAVS and for the production of type 1 IFN (Figure 2) [10–18]. To prevent its overactivation, RIG-I is actively targeted by many cellular factors that inhibit K63 ubiquitination (CYLD, USP1, and USP3) or tag it for proteasome degradation via K48 ubiquitin linkage (RNF122, RNF125) [19–23]. Other PTMs, such as acetylation (HDAC6) and SUMOylation (P1AS2*β*, TRIM38, and SENP2), or direct association of cellular proteins with RIG-I to disrupt its interaction with MAVS (NLRC5, NLRX1), have also been shown to contribute to RIG-I activation or repression. However, their overall contribution to the canonical phosphorylation-ubiquitination system remains to be elucidated [24–28]. Once activated, RIG-I and MAVS interact via their CARD domains to form prion-like aggregates that become the immune platform for the phosphorylation of IRF3/NF-*κ*B. This signaling relies on the recruitment of many regulatory subunits (TRAF2, TRAF5, TRAF6, and NEMO), which allows the phosphorylation of immune transcription factors via IKBKE, TBK1, and IKK protein kinases, leading to their nuclear translocation and the production of type 1 IFN with subsequent expression of ISGs (Figure 2) [29–34]. Based on sequence homology analysis, MDA5 and CARD-less LGP2 were identified as putative vRNA sensors (Figures 1(b) and 1(c)). The three proteins are collectively referred to as RLRs. Notably, these proteins have a similar helicase superfamily II (SF2) ATPase domain and CTD that will prove to be essential for their nucleic acid sensing function and distinguishing between different RNAs.

## 3. RLR Distinction of RNA Ligands

RIG-I and MDA5 are RNA helicases that survey the cytoplasm in search of PAMP (Figure 2). They have distinct but overlapping pathogenic RNA preferences, which enable differentiation of cytosolic self and nonself RNA. Initial studies in mouse embryonic fibroblasts deficient for MDA5 (MDA5−/−) showed that they can initiate an antiviral response when challenged with intracellular nonself RNA molecules containing a triphosphate moiety at the 5′ region (5′ppp) while RIG-I−/− cells cannot [35]. Moreover, when the 5′ region is capped or is treated with calf intestinal alkaline phosphatase to remove the phosphates, no stimulations are observed [36]. These findings gave the first evidence that RIG-I can recognize uncapped and phosphorylated 5′ RNAs while MDA5 could not. Subsequent studies showed that RIG-I is more likely to recognize short double-stranded RNA (dsRNA) molecules while MDA5 is activated by long dsRNA [12, 37, 38]. More recently, influenza U/A-rich 3′ regions of viral RNA segments were shown to activate RIG-I in a 5′ppp-independent manner via an unknown mechanism (Table 1) [39]. This recognition might be mediated by RIG-I's helicase domain instead of the paradigmatic CTD. Additional studies, such as examination of the crystal structures of the full RIG-I/MDA5 proteins bound to vRNA, are required to understand the fine molecular mechanisms related to the vRNA and the sensor structural properties, in order to have one unifying and comprehensive theory. Nevertheless, the physical characteristics of RLR ligands correlate exceptionally well with the type of viruses that are recognized by RIG-I, such as Sendai virus (SeV), vesicular stomatitis (VSV), influenza A (FLUA), and hepatitis C virus (HCV), and by MDA5, such as encephalomyocarditis virus (EMCV), norovirus, or murine hepatitis virus (MHV) (see Table 1) [39–41]. In short, RIG-I can recognize viruses that produce short and phosphorylated replication intermediates through its CTD, whereas MDA5 tends to recognize long vRNA molecules. Altogether, these data support the concept that cytoplasmic RNA helicases are sensors of nonself RNA and work together to ensure an optimal coverage of the full spectrum of viral nucleic acids, including replication intermediates and copy-back defective interfering (DI) genomes. Furthermore, these observations emphasize the importance of the 5′-triphosphate and dsRNA as molecular patterns that enable RIG-I/MDA5 to distinguish pathogenic from self RNA.

Now, how does the functioning of these RNA helicases differentiate self RNA from pathogenic RNA leading to the initiation of the RLR/MAVS antiviral signaling pathway? As discussed before, in resting cells, RIG-I and MDA5 are kept in a closed conformation (signal off) by their CTD. Upon contact with vRNA molecules, it is proposed that an ATP-dependent translocation along the dsRNA leads to the high-affinity binding with the CTD to expose the 2CARDs and to the promotion of the formation of stable RIG-I dimers [42–44]. Importantly, the ATP-dependent translocation was recently shown to contribute to the self versus nonself RNA recognition, as the ATPase/translocase activity removes RIG-I from abundant self RNA while locking it into the nonself RNA motifs following translocation, and binding it to the viral determinant such as 5′ppp, reducing background signaling and increasing sensitivity of vRNA detection [42, 44]. Following the recognition of the proper RNA ligand, RIG-I signaling is activated and cells enter an antiviral state characterized by the production of antiviral type 1 IFN and ISGs (Figure 2) [29–32, 34, 45]. If RIG-I binds to nonpathogenic RNA, it will be displaced by ATP hydrolysis to prevent the recognition of endogenous RNA and avoid unintentional signaling due to prolonged RNA binding. The lack of proper ATP hydrolysis by RNA sensors, such as RIG-I and MDA5, was recently linked to many genetic disorders whose pathogenesis is caused by an upregulated type 1 IFN signaling that leads to many autoimmune disorders such as Aicardi-Goutières syndrome (AGS), Singleton-Merten syndrome (SMS), systemic lupus erythematosus (SLE), and type 1 diabetes [46]. These disorders are caused by nonsynonymous point mutations located within the helicase/ATPase domain of MDA5 and RIG-I that confer a constitutive activation and implicate the aberrant sensing of nucleic acids for the inappropriate production of type 1 IFNs [47–50]. These studies emphasize the importance of a functional SF2 helicase domain for the discrimination of self and nonself RNA and the elicitation of an adequate and controlled immune response.

## 4. RNA Helicases as Sentinels for Cytoplasmic RNA and Antiviral Immune Responses

LGP2 is an RNA helicase, homologous in structure to RIG-I and MDA5, except that it lacks the 2CARDs that are required to initiate antiviral signaling via the MAVS adaptor protein (Figure 1(c)). Thus, LGP2 is not able to propagate the signal to produce type 1 IFN and must have a role that is different from RIG-I and MDA5 in the RLR pathway (Figure 2). Initially, LGP2 was proposed as a negative feedback regulator of the RLR pathway that would act by sequestering vRNA from RIG-I [51] or by displacing IKBKE from MAVS in order to terminate IRF3-dependent antiviral signaling [52]. Subsequent studies showed that CTDs of LGP2 and RIG-I are analogous and provided *in vitro* evidence that LGP2 CTD can interact with RIG-I to abolish its ability to initiate antiviral signaling [4, 53]. In addition, the latest study is reminiscent of the novel negative regulator of innate immunity KHSRP that associates with the CTD of RIG-I to maintain the receptor in an inactive state and attenuate its sensing of vRNA [54]. Upon viral infection, KHSRP competes with PAMP for the RNA recognition site located within RIG-I's CTD. This competition between KHSRP and vRNA is thought to be essential to maintain a proper activation threshold of RIG-I signaling and prevent unnecessary or disproportionate activation of the RLR pathway. Despite some initial controversies about its function in antiviral signaling, LGP2 is emerging as a sentinel sensor that cooperates with RIG-I and MDA5 to enhance their recognition of the vRNA substrate and to initiate type 1 IFN response against viruses such as ECMV and HCV (Table 1) [55–57]. According to this model, LGP2 can leverage upon its ATP-dependent/RNA helicase activity to assist and increase interactions of a larger subset of nucleic acid-derived PAMP with RIG-I or MDA5 and finally potentiate antiviral signaling. Additionally, it was recently shown that LGP2 inhibits a DICER-mediated processing of vRNA [58]. In contrast to the elaborated protein-based system found in mammals, plants and invertebrates rely on their RNA interference (RNAi) machinery to degrade vRNA and subvert viral replication [59]. This recent report provides evidence that LGP2 antagonizes the degradation of vRNA by DICER to keep the cytosolic PAMPs intact and allow their detection by RNA sensors. Further studies should provide key insights about the relationship between the antiviral RNAi system, LGP2, and the RLR pathway in mammalian cells. Interestingly, LGP2 sentinel function seems to be shared by many other DExD/H box RNA helicases such as DDX3, DHX9, DHX29, and DDX41, which bind directly to nucleic acids and interact with either RIG-I or MAVS to activate the pathway (see [60, 61]). Furthermore, RNA helicases from the Ski-2-like family have been described to act as sentinels for RIG-I activation and viral RNA degradation, as well as negative regulators of the RLR pathway. Indeed, SKIV2L teams up with exosomes to degrade RNA and limits activation of the RLR pathway upon activation of the unfolded protein response (UPR), and humans with a deficiency in SKIV2L have a type 1 interferon signature in their peripheral blood [62]. In this review article, we will concentrate on DDX60 and SNRNP200 to show the prototypical characteristics of a Ski-2-like helicase as a sentinel for cytoplasmic antiviral response. The DDX60 RNA helicase also acts as a cofactor of the exosome complex, which is involved in the degradation of various types of RNA molecules to maintain the quality of host RNA. However, upon viral infection, DDX60 acts as an ISG that helps cells to suppress viral replication by increasing interactions between vRNA and RIG-I/MDA5 to enhance antiviral signaling and type 1 IFN production [63]. DDX60 is also able to promote exosome-mediated degradation of HCV RNA (Table 1) that reduces cell stress from viral replication as a first line of defense, but in turn produces degraded vRNA agonists that are likely to be recognized by RIG-I/MDA5 and other sentinels in a feed-forward mechanism that enhances type 1 IFN production [64]. Overall, while additional studies are required to assess the role of DDX60 against many viruses and across different cell lines, the first insight into its mechanism of action highlights two important features of Ski-2-like RNA helicases as sentinel for cytoplasmic RNA: (1) they are able to detect vRNA and bring them to RNA sensors (RIG-I) to augment antiviral signaling by allowing for a more efficient detection of a cytoplasmic PAMP and (2) they are able to target vRNA to the RNA exosome, which turns them into immune-stimulatory molecules by revealing a molecular signature (e.g., short 5′ppp dsRNA) that can be recognized by RIG-I. More recently, we identified a novel sentinel, SNRNP200, a member of the Ski-2 RNA helicase family that is critical in the RIG-I/MAVS signaling pathway by promoting vRNA sensing and IRF3 activation via a direct interaction with TBK1 [65]. SNRNP200 is an essential member of the spliceosome complex along with several other RNA helicases that are responsible for removing introns from the pre-mRNA and give rise to coding mRNA [66–70]. Upon viral infection, SNRNP200 binds vRNA through its amino-terminal Sec 63 (Sec63-1) domain, relocates to the perinuclear region, and acts as an adaptor protein to potentiate IRF3 signaling. Much like other DExD/H box RNA helicases, SNRNP200 requires a functional ATPase/helicase activity in addition to a competent Sec63-1 domain of unknown function to promote IRF3-dependent IFN induction upon virus infection. Directed mutagenesis experiments further showed that a defective SNRNP200 C502A variant within the ATP-binding motif leads to constitutive type 1 IFN production *in vitro* [71], reminiscent of a phenotype of type 1 interferonopathies [46–50]. Thus, the immunoregulatory function of SNRNP200 recapitulates properties of RIG-I/MDA5 and sentinels; they all leverage upon their ATPase/helicase domain to unwind vRNA and detect and bind to a specific RNA motif as they translocate along the RNA strand, serving as scaffolding proteins to initiate antiviral signaling. This mode of action limits recognition of nonpathogenic RNA and the unnecessary activation of RLR signaling (as reviewed in [72]). In this perspective, it is reasonable to propose that antiviral RNA helicases are involved in the larger picture of RNA responsiveness, where they balance the need for innate defenses against pathogens and actively restrict involuntary RLR pathway activation.

## 5. Toll-Like Receptors (TLRs)

TLRs have an important role in recognizing molecular patterns associated with different pathogens. 11 TLR genes are present in the human genome, with TLR11 being a nonfunctional pseudogene. The majority of the TLRs are found on the plasma membrane, while TLRs 3, 7, 8, and 9 are present in the endosomal compartment [73]. Whereas those expressed on the cell surface predominantly recognize molecules of the microbial membrane, for example, proteins, lipids, and lipoproteins, endosomal TLRs detect viral, bacterial, or self nucleic acids. In this review we will focus on TLRs 3, 7, and 8 for their role in detecting extracellular RNA and viral particles [73].

## 6. TLR3 Expression and Ligands

TLR3 is expressed in the endosomes of immune cells, that is, monocytes, macrophages, dendritic cells (DCs) (other than plasmacytoid DCs), natural killer (NK) cells, T and B lymphocytes, mast cells, eosinophils, and basophils. Nonimmune cells, such as epithelial and endothelial cells, keratinocytes, fibroblasts, hepatocytes, astrocytes, and microglia, also express TLR3 [74, 75]. TLR3 recognizes dsRNA, the synthetic polyinosinic-polycytidylic acid (poly I : C), and polyadenylic-polyuridylic acid (poly A : U) (Table 1) [74, 75]. Moreover, TLR3 may be triggered by single-stranded RNA (ssRNA) with stable stem structures as described based on poliovirus RNA sequences [76]. However, further studies may be required to elucidate the exact mechanisms of such triggering.

TLR3 plays a significant role in the modulation of RNA and DNA virus-mediated innate immune responses. TLR3 senses dsRNA viruses such as members of the Reoviridae family including the rotavirus by sensing their genomic RNA; this recognition leads to the induction of inflammatory cytokines and type 1 IFNs [74, 77]. Moreover, TLR3 recognizes intermediate RNAs that are produced during the replication of other viruses such as the herpes simplex virus-1 (HSV-1), respiratory syncytial virus (RSV), West Nile virus (WNV), coxsackievirus B3 (CVB3), poliovirus, and influenza A virus (FLUA). The viral dsRNAs can reach the TLR3 in the endosomes upon phagocytosis of dying infected cells or by direct uptake from the medium by antigen presenting cells (Table 1) [74, 77]. The possibility of the presence of intermediate viral ssRNAs with stable stem structures as a reason for the detection of these viruses by TLR3, as observed in the case of poliovirus, remains to be investigated [76].

## 7. TLR3 Structure and Signaling Pathways

**Figure 3:**
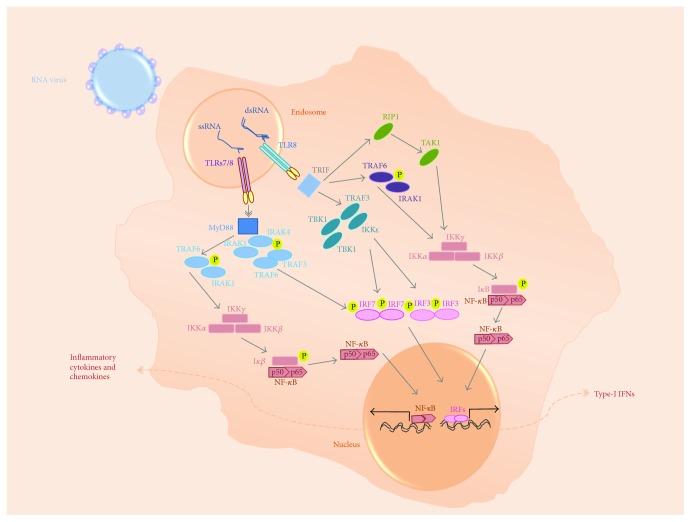
The pathways induced by TLRs 3, 7 and, 8. TLR3, TLR7, and TLR8 are expressed in the endosomes. The triggering through these molecules leads to the activation of NF-*κ*B and IRF signaling pathways, which results in the production of inflammatory cytokines and chemokines as well as type 1 IFNs.

TLR3 has a C-terminal cytoplasmic toll-interleukin 1 receptor (TIR) domain used for signaling, an N-terminal extracellular domain (ECD), and a single transmembrane alpha helix. The ECD has 23 leucine-rich repeats (LRRs); it is responsible for the binding of dsRNA (Figure 1(d)). The dimerization of ECDs initiates the signaling [74, 78]. The TIR domain-containing adaptor protein-inducing IFN-*β* (TRIF) is then recruited and undergoes slight conformational changes [79] to form a signaling complex together with TNF receptor-associated factor 6 (TRAF6), TRAF3, TBK1, IKK*ε*, and IKK (Figure 3). This leads to the activation of IRF3/IRF7 and NF-*κ*B, which results in the production of type 1 IFNs and inflammatory cytokines, respectively [74, 78].

In order to control the levels of inflammation induced by the triggering of TLR3, its signaling pathway is regulated by different molecules. Some act as positive regulators such as serine/threonine kinase receptor-associated protein (STRAP) that interacts with TBK1 and IRF3 [80], munc18-1-interacting protein 3 (Mint3) that stimulates the K63-linked polyubiquitination of TRAF3 [81], Src-associated substrate in mitosis of 68 kDa (Sam68) that may balance NF-*κ*B p65 and c-Rel activation [82], and finally S100A9 that acts during the early stages of TLR3 activation by easing the maturation of TLR3-containing early endosomes into late endosomes [83]. Other molecules act as negative regulators, such as Rho proteins that decrease the production of proinflammatory cytokines upon TLR3 triggering [84], SUMO-specific protease 6 (SENP6) that inhibits the NF-*κ*B-mediated expression of the proinflammatory genes [85], and miR-155 that controls TLR3 signaling by repressing molecules such as TAB2, IKK-*ε*, and RIP [86]. Interestingly, some oncogenic herpes viruses such as Kaposi's-sarcoma-associated herpes virus (KSHV) and Epstein-Barr virus (EBV) induce cellular miR-155 expression or encode the functional ortholog of miR-155, which might constitute a strategy to escape the immune responses induced upon TLR3 triggering [86]. In addition, several proteins in the TLR3 pathway are targeted by different PTMs, which also participate in the regulation of responses initiated by TLR3 triggering [6].

## 8. TLR3 and the Pathogenesis of Viral Infections

TLR3 has an important impact on the pathogenesis and outcome of several RNA virus infections. In fact, the level of expression of TLR3 is associated with the severity and outcome of HCV infection [87]. Moreover, single-nucleotide polymorphisms (SNPs) in the *TLR3* gene are associated with HCV-mediated liver disease progression and the development of hepatic fibrosis [88]. As mentioned above, TLR3 also plays an important role in establishing immune responses against HSV-1. Different studies showed that mutations in the *TLR3* gene are associated with the predisposition to HSV-1 encephalitis (HSE) in children [89–92] and adults [93, 94]. These mutations in TLR3 were shown to result in a lack of response to poly I : C and HSV-1 as observed in fibroblasts and induced pluripotent stem cell- (iPSC-) differentiated neural stem cells (NSCs), neurons, astrocytes, and oligodendrocytes [89, 90]. This impairment was characterized by the absence of production of IFN-*β* and IFN-*λ* in these cells [89, 90]. The association of mutations in the *TLR3* gene with varicella-zoster virus encephalitis was also shown [93]. Other studies have shown that TLR3 may influence the pathogenesis of RSV, CB3, and enterovirus 71 (EV71), severe fever with thrombocytopenia syndrome (SFTS), and HBV infections [95–99]. This highlights the important role played by TLR3 in the innate immune responses to viruses, although the exact mechanisms of recognition and how it is involved often remain elusive.

## 9. Targeting TLR3 in Antiviral Therapies and Vaccines

The potential use of TLR3 ligands in antiviral therapies and vaccines is suggested by different studies. For example, recently TLR3 ligands were shown to be efficient in reversing the latency of the human immunodeficiency virus (HIV) by the reactivation of HIV transcription in microglial cells [100]. Another study reported TLR3 ligands as candidates for anti-HIV immunotherapeutic strategies because these ligands increased the ability of HIV-infected DC to activate HIV-specific cytotoxic T lymphocytes [101]. TLR3 ligands were also shown to be potent adjuvants for vaccine preparations targeting influenza virus, HIV, and HSV-2 [102–104]. Interestingly, poly I : C derivatives (known as Ampligen) are potential adjuvants tested in vaccine preparations targeting influenza virus, HIV, and HPV [102].

## 10. TLRs 7 and 8: Expression and Ligands

TLRs 7 and 8 are expressed in the endosomes of a wide variety of cells including immune cells such as monocytes, macrophages, DC, and NK cells [105]. The expression of TLR7 is also reported in T and B cells [105, 106]. TLR8 is also expressed in mast cells and regulatory T cells [107, 108]. The expression of TLRs 7 and 8 is not restricted to immune cells, as they are also expressed in endothelial and epithelial cells, astrocytes, microglia, and hepatocytes, as well as tumor cells [109–111].

TLRs 7 and 8 share a lot of similarities, and recent findings suggest a potential compensatory role played by TLR8 in the absence of TLR7 [112]. TLRs 7 and 8 recognize guanosine and uridine- (GU-) rich or U-rich ssRNA sequences [113, 114]. However, we have shown that the presence of GU-rich sequences in ssRNA might not be sufficient, although necessary, to stimulate these TLRs [115]. In this study, several GU-rich sequences in the HCV genome were described; however, not all these sequences were able to trigger TLRs 7 and 8. In fact, the capacity of these sequences to trigger TLRs 7 and 8 was not influenced by their length or the number of GU repeats that they contain [115]. Interestingly, some cellular defense mechanisms that target vRNA may influence its detection by TLRs 7 and 8. In fact, the detection of phagocytosed vRNA by TLRs 7 and 8 is facilitated by the adenosine-to-inosine (A-to-I) editing, which is an important arm of the antiviral response [116]. Furthermore, 2′-O-methylation within an RNA sequence shapes differential activation of TLRs 7 and 8 [117, 118]. This modification leads to the triggering of TLR8 but not TLR7 by an RNA that was initially able to trigger both TLRs. The hypothesis that this might be due to a stronger binding by TLR7 than TLR8 will require further investigation. This change in the triggering leads to a different secretion of proinflammatory cytokines as it impairs IFN-*α* production but not IL-6 [118].

Because of the capacity to sense ssRNA, TLRs 7 and 8 have an important role in detecting RNA viruses and inducing antiviral immune responses. They can be triggered by viral GU- and U-rich ssRNA sequences, such as those in highly conserved untranslated terminal regions (UTR) of viral genomes that have a crucial role in viral protein translation and RNA replication [119]. The implication of TLR7 or TLR8 in detecting RNA viruses is different depending on the virus and the cell in which these TLRs are expressed. Viruses, such as yellow fever virus (YFV), rhinoviruses, and HIV, can be detected by both TLR7 and TLR8 [113, 120, 121]. However, the expression of TLRs 7 and 8 in a cell does not always guarantee their triggering by an RNA virus, even though the latter has RNA sequences that can be detected by these TLRs. This was shown in the case of the HCV genome, which has sequences that stimulate both TLRs 7 and 8 [115]. Nevertheless, the complete HCV particles do not induce responses through these TLRs in myeloid and plasmacytoid DC subsets and monocytes, whereas such stimulation takes place in macrophages without stimulating antiviral responses [115]. Differences in the ability of cells to detect an RNA virus via TLRs 7 and 8 were also described for Zika virus (ZIKV) infection, as no TLR7 activation was detected in primary human fibroblasts [122], while genes implicated in TLR7 and TLR8 pathways were found to be upregulated in the human neural progenitor cells (hNPCs) infected with this virus [123]. Moreover, some vRNAs are recognized by TLR7 but not by TLR8. This may suggest the presence of differences in the conditions that lead to the detection of ssRNA sequences by TLR7 and TLR8. For example, the measles virus (MV), Ebola virus (EV), dengue virus (DV), human T-lymphotropic virus type 1 (HTLV-I), and poliovirus are able to trigger TLR7 only, while the role of TLR8 in such recognition remains unclear (Table 1) [74, 124]. Nevertheless, SNPs in *TLR7* and *TLR8* genes were associated with immune responses to MV suggesting a role for both TLRs during MV infection [125].

## 11. TLRs 7 and 8: Structures and Signaling Pathways

TLRs 7 and 8 are single-pass transmembrane receptors composed of a pathogen-recognition LRR-containing ectodomain and a TIR domain [126]. TLRs 7 and 8 have 26 LRR motifs in their extracellular domain, which contain multiple insertions such as the Z-loop or undefined region situated between LRRs 14 and 15 (Figures 1(e) and 1(f)) [127]. Both TLRs are proteolytically cleaved in the endosomes at the level of the Z-loop by arginine endopeptidase and cathepsins, and the cleaved fragments are linked together [128]. This is essential for the dimerization and activation of these TLRs [129]. TLR7 and TLR8 dimers have a binding site for small chemical stimuli or degradation products of ssRNA and a second binding site that recognizes ssRNA oligonucleotides. Both these sites are required for ssRNA-induced activation [130, 131]. The TIR domains multimerize following the interaction of TLRs 7 and 8 with their agonists, which is important for the recruitment of myeloid differentiation primary response gene 88 (MyD88) [132]. MyD88 forms a complex with interleukin 1 receptor-associated kinase (IRAK) molecules. The pathway will eventually lead to the activation of transcription factors including IRF7 and NF-*κ*B, which will cause the production of type 1 IFNs and inflammatory cytokines, respectively (Figure 3) [132].

A number of molecules regulate TLR7 and TLR 8 signaling pathways and control the immune responses that are triggered upon stimulation of these TLRs. Some of these molecules are positive regulators such as UNC93B1, which physically associates with TLRs 7 and 8 and delivers them to endolysosomes [133]; hepatocyte growth factor regulated tyrosine kinase substrate (HRS) that is required for proper TLR7 trafficking to endolysosomal networks [134]; CCAAT/enhancer-binding protein beta (C/EBP*δ*) that enhances the transcription of TLR8 [135]; triggering receptor expressed on myeloid cells like 4 (TREML4) that enhances TLR7 signaling [136]; and pyruvate dehydrogenase kinase isozyme 2 (PDK2) that physically interacts with TRAF6 [134]. Spleen tyrosine kinase (Syk) was also shown as a positive regulator of the TLR7 pathway in the plasmacytoid DC (pDC) subsets. However, Syk may also negatively regulate the TLR7 pathway upon the stimulation of the regulatory immunoreceptors CD303 and CD85g in pDC, which suggests the presence of a dual role for Syk in the regulation of the TLR7 pathway [137]. Other molecules are also considered as negative regulators for the TLR7 pathway such as tripartite motif 35 (TRIM35) that stimulates the K48-linked ubiquitination of IRF7 [138] and SENP6 described above in the TLR3 section [85]. More studies are required to identify molecules that negatively regulate TLR8 signaling. Furthermore, different proteins implicated in the TLR7/8 pathway are subject to PTMs, which have a direct impact on the regulation of TLR7- and TLR8-induced responses [6].

## 12. TLRs 7 and 8 and the Pathogenesis of Viral Infections

TLRs 7 and 8 influence the pathogenesis and outcome of several RNA virus infections such as HCV. In fact, the spontaneous resolution of the HCV infection has been shown to be associated with a sustained hyperresponsiveness of pDCs and mDCs to TLR7/8 stimulation [139], and the clearance and progression of the HCV infection is modulated by variations in the TLR7 and TLR8 genes [140]. Moreover, the potential capacity of the vRNA of different influenza strains to stimulate TLRs 7 and 8 was found to be correlated to the virulence of the strains [141]. In addition, SNPs in the *TLR7* and *TLR8* genes were associated with the CD4 T cell count during an HIV infection [142] as well as the levels of type 1 IFN and proinflammatory cytokines and the progression to hepatocellular carcinoma during an HCV infection [143, 144]. Also, the low copy numbers of the *TLR7* gene is associated with the establishment of chronic HBV infection [145].

The triggering of TLRs 7 and 8 by viruses is not always an advantage for the immune system. HIV infection provides several examples for this phenomenon. In fact, TLR7 stimulation by the HIV ssRNA in CD4 T cells induces the anergy of these cells [146]. HIV requires the stimulation of NF-*κ*B upon the triggering of TLR8 to replicate in DCs [147]. In addition, HIV takes advantage of the cellular protein snapin that inhibits its detection by TLR8 in DCs to transinfect other cells [148]. In fact, inhibiting snapin expression leads to an increased localization of HIV-1 within the early endosomes that contain TLR8, the establishment of a proinflammatory response, and the inhibition of CD4 T cell transinfection [148].

## 13. Targeting TLRs 7 and 8 in Antiviral Therapies and Vaccines

TLR7 and TLR8 ligands are potential candidates for antiviral therapeutic and vaccine strategies. Hence, the capacity of TLR7 and TLR8 ligands to inhibit HIV replication and to activate the HIV reservoir is being investigated [149, 150]. Moreover, TLR7 and TLR8 ligands were proposed to be used as adjuvants in FLU vaccine preparations [151]. Furthermore, the TLR7 agonist Imiquimod (R-837 or trade name Aldara) and TLR7/8 dual agonist Resiquimod (R-848) are topical treatments for HPV-induced warts [102]. Although systemic administration of Imiquimod may be highly toxic, Resiquimod showed promising results as an adjuvant in an anti-HSV trial [102].

## 14. Conclusion and Perspectives

Up to this point, we have established the key players and mechanisms of the antiviral innate immunity protecting the host from RNA viruses. We have shown that RNA helicases and TLRs 3, 7, and 8 are essential nucleic acid sensors that survey the cytoplasmic and endosomal spaces for extracellular threats and, upon engagement, elicit type 1 IFN responses to restrict viral replication. Recent findings showing the involvement of unconventional PTMs, such as SUMOylation and acetylation, to the regulation of these PRRs have cleared the way to a better understanding of antiviral signaling, host-factor interactions, and the etiology of various autoimmune diseases. Further studies using a system-based approach, similar to the one used to identify SNRNP200 and KHSRP, together with the understanding of the nature of ligands and inhibitors of PRRs should provide additional knowledge to identify novel approaches for treatments and vaccine preparations directed against RNA viruses and beyond, in autoimmune diseases and cancers [102, 152]. Moreover, the potential ability of RNA viruses to interfere with the mechanisms regulating the signaling of these PRRs in order to escape detection necessitates more investigations. Additionally, with the description of a myriad of novel host factors involved in RLR signaling, one might wonder which components (RNA sensors, sentinels, positive, and negative regulators) are required for the minimum or optimal antiviral response, and what are the differences in this hierarchy according to cell type or pathogen. There is a coordination between TLRs and RLRs, as seen in some autoimmune diseases and viral infections [153–156]. The mechanisms that control this cooperation in detecting RNA viruses, and the consequences of such collaboration, deserve to be investigated in more depth. Lastly, PRR-targeting therapies have gained great momentum in the field of cancer immunotherapy. Recent reports have shown that RIG-I activation can induce tumor cell death directly via the production of IFN, or indirectly via the activation of cytotoxic CD8 T cells and NK cells, and via DC-mediated antigen cross-presentation of tumor-associated antigens to CD8 T cells [68]. In addition, the modulation of TLR3 and 7 can be leveraged as anticancer therapies since their signaling can increase cytotoxic T cell activity and directly induce cancer cell death via apoptosis, pyroptosis, and autophagy. Thus, the recent advances in our understanding of innate antiviral immunity have clearly given a new momentum towards the development of therapeutic agents targeting PRR for infectious diseases and cancers. These strategies are in the preclinical or early clinical phase such that it is still unknown if these PPR-targeting agents will translate into effective, safe, and tolerable anticancer therapeutics.

## Figures and Tables

**Table 1:** RNA viruses and ligands recognized by RLR and TLR.

RNA sensor	RNA preference	Representative viruses
*Helicases*		
RIG-I	Uncapped 5′ and phosphorylated ssRNA, short dsRNA, and U/A-rich 3′ regions of viral RNA	Adenovirus, DENV, EBOV, FLUA/B, HCV, HSV, JEV, LACV, LASV, MV, NDV, NV, PIV5, Reoviridae, RSV, RV, RVFV, SeV, VSV, and WNV
MDA5	Long dsRNA	Adenovirus, DENV, EBV, ECMV, enteroviruses, HCV, HSV, JEV, MV, NDV, norovirus, NV, PIV5, Reoviridae, RSV, SeV, SAFV3, TMEV, and WNV
LGP2^∗^	dsRNA	ECMV and HCV^∗^
DDX60	dsRNA	HCV, RSV, and VSV
SNRNP200	dsRNA	FLUA, HCV, and SeV
*TLRs*		
TLR3	dsRNA	CVB3, HSV-1, poliovirus, Reoviridae family (rotavirus), RSV, and WNV
TLR7	GU- and U-rich ssRNA	DENV, EBOV, FLUA, HCV, HIV, HTLV-I, MV, poliovirus, rhinoviruses, and YFV
TLR8	GU- and U-rich ssRNA	FLUA, HCV, HIV, rhinoviruses, and YFV

CVB3: Coxsackie B virus; DENV: dengue virus; EBOV: Ebola virus; EBV: Epstein-Barr virus; ECMV: encephalomyocarditis virus; FLUA: influenza A virus; FLUB: influenza B virus; HCV: hepatitis C virus; HIV: human immunodeficiency virus; HSV: herpes simplex virus; JEV: Japanese encephalitis virus; LACV: La Crosse virus; LASV: Lassa virus; MV: measles virus; NV: Nipah virus; PIV5: parainfluenzas virus 5; RSV: respiratory syncytial virus; RV: rabis virus; RVFV: Rift Valley fever virus; SAFV3: Saffold virus 3; TMEV: Theiler's virus; SeV: Sendai virus; VSV: vesicular stomatitis virus; WNV: West Nile virus; YFV: yellow fever virus. ^∗^More studies are required to clarify the capacity of LGP2 to detect viruses including ECMV and HCV.
